# Cannabidiol treatment in an adolescent with multiple substance abuse, social anxiety and depression

**DOI:** 10.1007/s40211-020-00334-0

**Published:** 2020-02-12

**Authors:** Clarissa Laczkovics, Oswald D. Kothgassner, Anna Felnhofer, Claudia M. Klier

**Affiliations:** 1 Department of Child and Adolescent Psychiatry, Medical University of Vienna, Währinger Gürtel 18–20, 1090 Vienna, Austria; 2 Department of Pediatrics and Adolescent Medicine, Medical University of Vienna, Währinger Gürtel 18–20, 1090 Vienna, Austria

**Keywords:** Adolescent, Cannabidiol, Anxiety, Depression, Substance use disorder, Adoleszenz, Cannabidiol, Angst, Depression, Substanzmißbrauch

## Abstract

In this report, we present a case of a 16,9-year-old patient with multiple substance use disorder (cannabis, MDMA, cocaine, ecstacy), severe depression, social phobia and narcissistic personality disorder.

We administered Cannabidiol (CBD) capsules in different dosages (starting dosage 100 mg up to 600 mg over 8 weeks) after unsuccessful treatment with antidepressants.

CBD was a safe and well tolerated medication for this patient. Upon treatment with CBD and cessation of the antidepressant medication, the patient improved regarding depressive as well as anxiety symptoms including simple phobias and symptoms of paranoia and dissociation. Furthermore, the patient quit abusing illegal drugs including THC without showing withdrawal symptoms. This is the first report of CBD medication in a patient with multiple substance use disorder with a positive outcome.

Until today it is not clear if CBD holds promise as a therapeutic option in substance use disorder as RCTs are lacking, but in this single case the substance seems to work in various domains.

## Abstract (de)

Wir präsentieren den Fall eines 16,9 Jahre alten Patienten, der aufgrund eines multiplen Substanzmissbrauches (Cannabis, MDMA, Kokain, Ecstacy), schwerer depressiver Symptomatik, einer Sozialphobie und einer narzisstischen Persönlichkeitsstörung behandelt wurde.

Wir verschrieben Cannabidiol (CBD) in Kapselform in unterschiedlichen Dosierungen (100 mg bis 600 mg pro Tag über 8 Wochen), nachdem die antidepressive Medikation keine Symptomreduktion brachte.

CBD wurde zu allen Zeiten gut vertragen. Auch nach Beendigung der antidepressiven Medikation profitierte der Patient bezüglich der depressiven Symptomatik, der Angstsymptomatik sowie paranoider und dissoziativer Symptomatik. Außerdem konsumierte der Patient während der Behandlung keine weiteren Substanzen wie THC oder andere illegalen Drogen, ohne dass es zum Auftreten von Entzugserscheinungen kam.

Es ist noch nicht erwiesen, ob CBD eine alternative Therapieoption bei Patienten mit multiplem Substanzabusus darstellt, da keine randomisiert-kontrollierten Studien publiziert sind. Bei unserer Fallstudie konnten wir eine Wirksamkeit in verschiedenen Bereichen der Psychopathologie beobachten.

## Introduction

Cannabis sativa contains more than 100 phytocannabinoids; one of them, ∆9-tetrahydrocannabinol (THC), is considered responsible for the psychotropic effects, known as a “high” as well as for diverse side effects, for example anxiety and/or panic attacks, altered body image, auditory and/or visual illusions and pseudohallucinations.

One other major component, cannabidiol (CBD), attenuates the psychotomimetic and anxiogenic side effects of THC [1] and is, thus, thought to contain anxiolytic and antipsychotic properties.

First randomised controlled trials (RCTs) among psychiatric populations predominantly focused on patients with psychotic disorders [2–4], who mostly showed significant improvement, as well as on patients with ultra-high risk for schizophrenia [5], with less anxiety and paranoia in the CBD group.

In contrast, only little is known about the effect in patients with anxiety disorder [6–10]. Similarly, regarding substance use disorders, there are only few studies on patients with cannabis dependence indicating a larger reduction of withdrawal symptom severity when compared to placebo [11–13]. Data pertaining to multiple substance use disorder and psychiatric comorbidities are still lacking. The reader is referred to the literature for a recent review on CBD and psychiatric and mental disorders [14, 15].

In the hitherto published data, CBD was mostly well tolerated with side effects similar to placebo except for mild sedation [14].

## Case

In this report, we present a case of a patient with multiple substance use disorder, severe depression, social phobia and narcissistic personality disorder according to ICD-10 criteria. The diagnosis of personality disorder was based on clinical observation as well as the STIPO Interview [16] for diagnosing the structural level of personality organization, which indicated a borderline structure with predominantly narcissistic personality traits.

We administered CBD capsules in different dosages (starting dosage 100 mg and increasing up to 600 mg) after ineffective treatment with antidepressants (sertraline) for over 6 months.

The 16.9-year-old male patient sought help at a large, urban and public clinic in December 2018. He presented with the symptoms of paranoia, derealization, attention deficit, severe depression, social anxiety and social withdrawal as well as multiple substance abuse. At his first appointment, he was using THC on a daily basis and indicated to consume MDMA, cocaine and ecstasy once a week. He had a history of psychiatric treatment over the last 2 years and had been treated with sertraline 100 mg administered orally once daily for 6 months. No other medication was given to the patient.

After written informed consent was provided by the patient and his legal guardians, we started the administration of CBD with an initial dosage of 50 mg twice daily, in the morning and evening. Within 3 weeks, we increased the dosage gradually to 300 mg twice daily (TD 600 mg/day), evaluating adverse effects and clinical improvement. The patient was treated in a day clinic setting, where he received group therapies and individual psychotherapy as well as social cognition training, occupational therapy and physiotherapy. The patient’s parents had support via weekly meetings with the treating psychiatrist.

Cannabidiol was well tolerated at all times and the patient showed good adherence. By his request, he discontinued sertraline after 3 weeks of CBD treatment from one day to the other. Prior intake of sertraline was documented by drug monitoring. Plasma levels were 84.9 ng/mL (therapeutic range 10–150 ng/mL). Sertraline was initially chosen because of the predominant symptoms of social anxiety und depressive mood. Clinically, he showed no difference in mood and anxiety symptoms after abrupt cessation of the antidepressant medication.

There were no side effects regarding heart rate, blood pressure, and weight. Urine drug screenings performed once a week revealed no further usage of chemical drugs. Screenings were positive for THC as known for chronic THC abuse and turned mostly negative after 6 weeks of treatment. Routine blood analysis showed normal values for blood count, liver enzymes, electrolytes, kidney function and inflammatory enzymes. The patient reported no side effects.

## Psychological assessments

We evaluated symptoms of depression, anxiety and early psychosis using a pre–post design. Consequently, the patient was assessed prior to CBD administration and after 8 weeks. Moreover, depressive symptoms were measured twice after 4 weeks and 8 weeks respectively. We used the Beck Depression Inventory II [17] and the German version of the Fear Survey Schedule for Children—Revised [18] to assess depression and anxiety symptoms. The short screening Checklist of the Early Recognition Inventory [19] was used as screening instrument for risk of psychosis. Additionally, the German version of the Wechsler Intelligence Scale for adults [20] was used for the measurement of cognitive functions at baseline level. Results are presented in Table 1.

**Table 1:** Development of symptomatology during CBD treatment

	T1 (baseline)	T2 (4 week follow-up)	T3 (8 week follow-up)
	Before CBD onset	After stopping sertraline	Stable CBD treatment^a^
**Depressive Symptoms**			
*Raw scores (cut-off label) of the BDI-II*^*b*^	22 (Moderate depressive episode)	10 (Minimal depressive episode)	7 (Minimal depressive episode)
**Anxiety Symptoms**			
*Raw scores (T values) of the German FSSC‑R*^*b*^	52 (60)	–	31 (50)
Fear of Danger and Death^b^	7 (50)	–	4 (45)
Fear of Separation	5 (45)	–	4 (45)
Social Anxiety^b^	13 (70)	–	7 (60)
Fear of the Unknown^b^	13 (70)	–	9 (60)
Fear of Animals	2 (50)	–	3 (55)
Medical Fears^b^	7 (65)	–	3 (50)
Fear of Evaluation and School Anxiety^b^	5 (55)	–	1 (45)
**At-risk mental state**			
*Raw scores of the ERIraos*^*b*^	12	–	4
Mental disorder ^b^ (affective symptoms, withdrawal, school and sleep problems)	4	–	0
At-risk psychosis ^b^ (rumination, social insecurity, paranoia, special interests, tension)	5	–	2
Beginning psychosis (depersonalisation, derealisation, symptoms of schizophrenia)	3	–	2

^a^Stable medication with CBD, no other psychopharmacological treatment applied

^b^Show clinically relevant improvement

## Summary and conclusion

In this case, CBD was a safe and well-tolerated medication for a patient with multiple substance abuse, social phobia, depression and a comorbid personality disorder. Upon treatment with CBD and cessation of the antidepressant medication, the patient improved regarding depressive as well as anxiety symptoms including simple phobias and symptoms of paranoia and dissociation. Furthermore, the patient stopped abusing illegal drugs including THC without showing withdrawal symptoms. The absence of illegal drug intake was assessed via daily clinical evaluation and confirmed using urine tests showing continuously negative results for a broad range of substances.

To date, two RCT studies have been published regarding cannabis dependency and withdrawal using a combination of CBD and THC [11, 12]. Allsop et al. [11] used a dosage of max 86.4 mg of THC and 80 mg of CBD/day. They reported a reduction of the withdrawal symptoms in the treatment group including irritability, depression and craving. Similarly, Trigo et al. [12] used a combination of THC and CBD (up to a dosage of 108 mg THC/100 mg CBD) and their findings replicated the efficacy with regards to withdrawal symptoms, but not regarding craving [12]. In a follow-up study, the authors found no significant differences between the treatment vs. placebo group on withdrawal scores [13]. In comparison, one case of a young adult with cannabis addiction was treated with CBD oil only with good results in abstinence, anxiety and sleep [21].

In sum, however, empirical evidence regarding multiple substance abuse and psychiatric comorbidities is still lacking. Bhattacharayya et al. [5] showed that after administration of 600 mg/day of CBD in patients with ultra-high risk presented with significantly less paranoia and anxiety compared to the placebo-treated patients. Hence, we decided to use greater dosages of CBD only, reflecting on the data for individuals with ultra-high risk for psychosis, since our patient presented with these symptoms additionally to substance use disorder. The rapid improvement of paranoid symptoms and dissociative symptoms may also be due to cessation of cannabis usage while in treatment in the day clinic setting. Improvements in depressive and anxiety symptoms, especially social anxiety and withdrawal, were seen gradually while increasing the CBD dosage. Since the patient reported good tolerability and no adverse effects, we increased the dosage to 600 mg/day. Concerning antipsychotic properties of CBD, Leweke et al. [2] reported clinical improvement at similar CBD dosages (600–800 mg/day). This improvement was significantly associated with elevated anandamide serum levels, reflecting a possible mode of action [22]. Cannabidiol does not activate cannabinoid receptors, but moderately inhibits the degradation of the endocannabinoid anandamide. Further research should consider examining serum anandamide levels.

To date, it is not clear whether CBD holds promise as pharmacotherapy for substance use disorder as RCTs are lacking and further investigation into CBD’s mechanism of action and its efficacy are warranted.
